# Clinical Experiences with Ascher’s Artificial Enamel

**Published:** 1910-05-15

**Authors:** Edgar F. Lewis

**Affiliations:** Rochester, N. Y.


### CLINICAL EXPERIENCES WITH ASCHER'S ARTIFICIAL ENAMEL.

BY EDGAR F. LEWIS, D.D.S., ROCHESTER, N. Y.

In doing my little bit this evening toward helping the society and helping each other, I want to say first of all, that I have helped myself the most.

This may seem and no doubt is somewhat selfish on my part, but I couldn't help it, for the preparation of this paper was thrust upon me by the committee that has to do with furnishing the writer's programme.

Did you ever look for a word in the dictionary and before your eyes would light upon it and as you turned the pages, in spite of yourself you would read half a dozen other things that interested you and that you would like to know more about?

That is exactly what happens when one undertakes to write a paper. So in listening to what I have to say you may be reminded of the young lady who said she didn't care to read Shakespeare's works, they were so full of quotations.

In order that you may know which side of this enamel fence I am on, I wish to say that I am now getting excellent results with Ascher's Artificial Enamel and like it very much. I have had my troubles with it, to be sure, and felt more than once like giving the whole thing up for a bad job. But I became determined to succeed. That is to say because I had had some successes with it I felt that it was a wonderful filling material if I could only learn how to always make a good filling with it.

You see I had been through the Porcelain Campaign and knew what the troubles were in that line, and though I had had some failures I had also had some beautiful successes, but only after the utmost diligence and painstaking.

So I set about to remedy my enamel failures and if possible overcome the possibility of a failure. I dreaded the thought of returning to gold in the anterior teeth with all of its labor, detail, unsightliness and imperfections.

I am going to relate my experience with one patient, a friend of mine. This gentleman had six large approximal cavities, not involving the corners in his anterior upper incisors. They had been filled with gold but needed refilling. I suggested the enamel and after some explanation, and in three sittings I thought I had done a pretty nice looking piece of work. In a few weeks they commenced to darken and before a great while discolored so badly I concluded to remove them.

Mr. Pinches was in town at this time and I got in touch with him and explained the case to him.

We decided to remove them and as arranged, Mr. Pinches was on hand to view the remains which were as black as your hat. They seemed to have even darkened the floor of the cavity. All was removed and put in shape to our satisfaction, every vestige of discolored dentine cut out. I remember Mr. Pinches being so particular about that point. For fear that my filling material might have become contaminated by hook or crook, Mr. Pinches used his own, a supply of which he had with him.

If you please, he mixed it himself and when he said the word I inserted the filling, two of which were in this way replaced.

But gentlemen, it may amuse you to know that my patient was patient enough to have them removed three months later.

I declare I was disheartened and discouraged but with more determination than an Irishman went at them again. The Irishman said when asked about the people he sprung from "I sprung from nobody, I sprung at 'em."

So I sprung at 'em again the idea being this time to flow an oxyphosphate cement over the floor and walls of the cavity.

As far as I remember, discoloration seemed to be the only thing I had trouble about. If I had other troubles, I attributed them to faulty manipulation in some detail. So the six fillings were replaced for the third time and after several months I find one of them slightly discolored in one corner. It looks to me as though I hadn't quite covered the entire floor with the oxyphosphate cement. The others are in excellent condition and the gentleman is well pleased with them.

For the benefit of those who may not have read The *Dental Summary* of last November I will read what one or two of our foremost dentists say regarding Silicate Cements.

Dr. J. R. Callahan, Cincinnati, Ohio, says: "I have been using Silicate Cements in an experimental way since they were first put on the market. Have seen no recurrent decay or leakage. The cavity walls should be protected by cement or varnished lining. My fillings have shown no discoloration. I do not, however, consider them safe practice unless the patient understands that they are expensive and may have to be removed from time to time.

Dr. J. P. Root, Kansas City, says: "I used them for about a year and ceased about eight months ago. I do not consider them permanent except in a very limited degree. Nearly all failures were due to discoloration. I have examined the edge and surface conditions to my sorrow for they were disastrous."

Dr. Joseph Head, Philadelphia, says: "My experience with Silicate Cements has been so poor that I have found them of little value except as a temporary filling, — it is true that sometimes they last well.

Dr. H. B. Tileston, Louisville, Kentucky, says: "I have been using Silicate Cements for four or five years. As to their solubility in the oral fluids, I consider them permanent. My failures have been due to one or more of several causes, the fault lying slightly with myself and not with the material, except in using a make that in itself was a failure. I have observed some discoloration under the labial enamel plate in incisors, which seem to indicate wall leakage, but which might be due to imperfect adaptation of the cement to the under surface of the enamel or to some carious matter left there or possibly to some stain left from a previous leaky metallic filling. In deep cavities I generally use some material like oxyphosphate."

So you see, gentlemen, that it has become quite a general practice to use the cement before the enamel.

I hope sometime to find out why the fillings do not discolor when they are properly cemented in. Doesn't it seem as though the discoloration came from the bottom instead of the surface?

While the magazines are full of directions, not to mention the directions that come with each package, yet we cannot help but have a certain individuality in our work to the extent at least of putting great value on some detail which may seem insignificant to another operator, and if you are so fortunate as to hit upon all of the valuable details to the exclusion of the unimportant ones you are fortunate indeed.

Some of the articles I have read are positively pathetic, for according to their own writings they have failed utterly to make a good filling with so good a filling material as Ascher's Artificial Enamel. While his next door neighbor testifies to his wonderful successes.

But I must get to my own testimony. First of all I have limited its use (with one or two exceptions) to labial or proximal cavities in anterior teeeth.

One of the exceptions was a distal and occluding cavity of a lower left bicuspid which I put in for Mr. Pinches, which, he wrote me recently, is in perfect condition. The other exception was an upper bicuspid distal occluding which I put in a couple of years ago and which I saw last week. It too was in fine condition with absolutely no discoloration.

The rubber dam is adjusted on a sufficient number of teeth to have plenty of room to work and see well what you are doing. That little thing alone always seemed to me to be very important. I saw last week what was left of four Ascher's Enamel fillings which had been put in about a month before without the rubber dam. Would anybody here think of doing such a thing. The instructions were evidently not followed closely.

It is needless to tell you how to prepare the cavity for I am sure you all know. Be sure the cavity is absolutely clean. To make sure of this very important detail I wash out the cavity and surrounding parts with alcohol. I always light the saturated cotton of alcohol over a flame to take the penetrating chill off. This little act of kindness or Christianity, which ever you choose to call it, is very much appreciated by the patient.

The next thing of importance is one of the important things I do not do. I do not use anything to sterilize the cavity such as an essential oil or carbolic acid, for the reason that it might have something to do with discoloration. I don't know whether it does or not but I prefer not to take any chances.

As to the color to select be sure and choose it before putting on the rubber dam. In case you are puzzled as to which of two shades to use, I think it best to use the darker one. Rather than depend on the shade guide, I have mixed a lump of each shade in stock and mounted it on the corner of one of my cards. I have several which were made by mixing two colors together in different proportions.

It is best to mix the powders as best you can while they are dry. I have always used the same glass slab and the same spatulas for this work. Just what value to attach to this I am not prepared to say for if you would use any clean slab and any clean spatula you will probably get just as good results, but be sure they are clean. Some of you may have noticed how tenaciously the last vestige of enamel will cling to the glass. Try the use of fine pumice, water, and a rag, it will remove it like a flash. I am going to have that patented, incorporate a company and sell some stock, for it is the only thing I ever invented.

I arrange two slabs side by side with powder and liquid on them ready for use. First I mix the oxyphosphate and flow a thin film over the floor and walls of the cavity. I use Klewe & Co's Porcelain Inlay Cements which are very finely ground and when mixed quite thin do not leave a line of demarkation between the enamel filling and the tooth and besides it sets slowly and gives plenty of time to mix the enamel.

When I flow the cement over the floor of the cavity I am always reminded of my professor of operative dentistry the late Dr. John I. Hart, who would talk about flowing cement.

I have always found it a very hard thing to do, indeed it is often difficult to get the cement to the bottom of a cavity without having it run up the instrument or dragging it off on the margins. In this work if your cement was inclined to set quickly or stiffen quickly you would never succeed in getting the walls covered as I have discovered when getting it a trifle thick.

I mix the enamel and putting a little of it on the point of a metallic instrument which I like very much for the purpose, I pack it quickly. Then with enough enamel to more than fill the balance I bend a celluloid strip over it and draw down quickly and remove instantly so as not to let it stick, at the same time hugging it tightly, especially at the cervical margin for there is where it is hard to finish, especially if the teeth are close together.

It is a good idea to wipe a thin film of vaseline on the celluloid strip to prevent it from sticking.

Just here let me caution you not to fuss with it after it is packed and drawn into shape. I always wait not less than ten minutes before commencing to finish it. That gives both cements time to crystallize pretty well, and will overcome the possibility of dislodging it or injuring the margins. To finish and polish I use the cuttlefish disks one-half inch regular grit and one-half inch fine grit, which is really no grit at all. I have tried the celluloid disks but don't like them for fear of cutting the rubber dam. For final finish I draw a smooth strip across a cake of paraffine, then vigorously use it on the filling.

Finally I cover the filling with liquid court plaster or its equivalent called Cavatini, which is sold at the dental depots. This will dry quickly, but a blast of warm air will help. In removing the rubber dam I first cut it where it goes between the teeth so as not to peel off the varnish.

As no vaseline is used it is of course unnecessary to wipe off the filling with alcohol.

In the original directions where vaseline was advised, wiping off the filling with alcohol was a part of the program. To me that always seemed like a dangerous thing to do, especially after great care had been taken to keep away moisture of any kind.

I keep a strict record of the shade used as well as the color of the cement, so at a later date if I wished to fill an adjacent tooth I would know whether to use the shade I used before or another.

